# ﻿*Hypericumliboense* (Hypericaceae), a new species from Guizhou, China

**DOI:** 10.3897/phytokeys.237.110482

**Published:** 2024-01-12

**Authors:** Tian-Rou Wu, Jian Xu, Ming-Tai An, Jiang-Hong Yu, Feng Liu, Zheng-Ren Chen

**Affiliations:** 1 College of Forestry, Guizhou University, Guiyang 550025, Guizhou, China; 2 Guizhou Botanical Garden, Guiyang, 550000, Guizhou, China; 3 College of Life Sciences, Guizhou University, Guiyang 550025, Guizhou, China; 4 Guizhou Maolan National Nature Reserve Administration, Libo 558400, Guizhou, China

**Keywords:** Molecular evidence, morphology, phylogeny, taxonomy

## Abstract

*Hypericumliboense* M.T.An & T.R.Wu, **sp. nov.** (Hypericaceae) is a newly described species found in the Maolan National Nature Reserve of Guizhou Province, where it grows in rocky habitats without soil on karst mountain tops. In this study, key morphological characters were compared between the new species and the other known *Hypericum* species of Hypericaceae. DNA sequences were extracted from the leaves of the new species, with nuclear gene sequences (ITS) generated to reconstruct phylogenetic trees and describe its phylogenetic position in relation to other species of *Hypericum*. Our results show that the proposed new species has the typical characteristics of the genus *Hypericum* in morphology being similar to *Hypericummonogynum*, but differing in its sessile and semi-clasped leaves, long elliptical to long circular leaf blades, thickly papery to thinly leathery, with entire and wavy leaf margins. The abaxial side of the leaves is covered with white powder, giving them a grey-white appearance. The main lateral veins of the leaves are 8–15-paired, and the midvein on both sides is convex. The main lateral veins and midvein branch are conspicuous, with tertiary venation forming a network on the leaf surface and appearing prominently sunken. The inflorescences are 1–3-flowered, with a large calyx and conspicuous veins. The molecular phylogenetic analysis (PP = 1.00) provided substantial evidence for the proposition of *H.liboense* as a new species within *Hypericum*. Morphological and molecular evidence is presented, corroborating the proposition of the new species, including a comprehensive account of the distinctive morphological attributes of *H.liboense*, along with its key distinguishing features from similar species.

## ﻿Introduction

*Hypericum* L. is the largest genus of the family Hypericaceae, with approximately 470 species worldwide (Dauncey et al. 2019), especially in temperate regions of the Northern Hemisphere and on tropical high-altitude mountains (Crockett and Robson 2011). In China, the genus is known to include a total of 68 species and nine subspecies, of which 33 species are endemic to the country. About 46 species and four subspecies are abundant in the western and southern regions of China, with few distributed in Xinjiang (Robson 2012). *Hypericum* plants are mostly herbs or shrubs, less often trees, and the flowers are often yellow or golden and occasionally white. Some species of this genus are cultivated around the world due to ornamental value, while some species have high medicinal value (Galeotti 2017; Bertoli et al. 2018; Marrelli et al. 2020; Revuru et al. 2020; Caldeira et al. 2022; Fang et al. 2023; Luo et al. 2023; Zheng et al. 2023).

The genus *Hypericum* was originally classified under the family Guttiferae based on morphological studies (Bessey 1915). However, the morphological characteristics of plants grown in different regions vary widely, leading to controversy among scholars regarding the main morphological basis for species identification. The rapid development of molecular technology (ITS), particularly the emergence of molecular marker technology, has provided compelling evidence for the study of classification, genetic relationship and developmental position of many plants (Yu et al. 2022; Deng et al. 2023; Ya et al. 2023), and the construction of phylogenetic trees is widely employed to demonstrate genetic relationships among species in the classification of *Hypericum*.

The APG IV system (The Angiosperm Phylogeny Group 2016) split the broad Guttiferae family into three families, namely Guttiferae, Hypericaceae, and Calophyllaceae, based on molecular evidence. Furthermore, the family Hypericaceae was divided into three tribes and ten genera (including the genus *Hypericum*). Robson (1972, 1977, 1985, 1990, 2001, 2006, 2010a, 2010b, 2012, 2016) has provided detailed monographic and molecular phylogenetic assessments of *Hypericum* and has classified the genus into 36 sections and 469 species. Nürk et al. (2015) utilized ITS sequence to establish the phylogenetic relationships within Hypericum ; their findings demonstrated that sect. Ascyreia was non-monophyletic. It was exhibited as a division into two sects: eastern *Ascyreia* and western *Ascyreia* (Meseguer et al. 2013). The completion of the taxonomic part of the *Hypericum* provides not only a taxonomic baseline and valuable tool for the identification of taxa, but also a rich resource for research into many other aspects of the biology and evolution of the genus. However, relationships between the more complete branches in *Hypericum* remained unresolved, and the classification problem of *Hypericum* still needs further in-depth research.

In 2022, we participated in a plant survey in a karst area of the Maolan National Nature Reserve in Guizhou, China, and discovered an unusual specimen of Hypericaceae. After field investigation and collection of specimens, we conducted detailed morphological analyses and realised that the morphological characteristics of this species were similar to those of *Hypericum*, but there were obvious differences in the leaf and calyx from the species occurring in China. To effectively differentiate species *H.liboense* from others within the genus *Hypericum*, this study utilized phylogenetic analysis based on morphological identification and description, combined with ITS sequences. As a result, a conclusion was reached, designating it as a novel species within the realm of scientific understanding.

## ﻿Material and methods

### ﻿Phylogenetic analysis

The ITS sequence, a highly reiterated tandem sequence in the nuclear genome, exhibits rapid changes, providing abundant variation and informative sites (Meseguer et al. 2013; Nürk et al. 2015). This sequence also demonstrates the highest level of species resolution accuracy (Chinese Plant Bol Group et al. 2011). In this study, we extracted DNA sequences from fresh leaves of *H.liboense*, followed by PCR amplification and instrument detection to obtain ITS sequences. A total of 55 *Hypericum* species were included in the analysis dataset, with ITS sequences obtained from NCBI (https://www.ncbi.nlm.nih.gov/). These 55 species represented 34 taxa of the genus *Hypericum* (Table 1), with 1–10 species selected as representatives for each taxon (Nürk et al. 2013). However, species of sect. Umbraculoides and sect. Thasia were not included in the analysis due to lack of ITS sequence data. *Thorneacalcicole* Standl. & Steyerm was used as the outgroup (Park and Kim 2004).

**Table 1.** Information of samples used for phylogenetic inference in this study.

Section number	Specie number	Species	GenBank No.	Section
1	1	*Hypericumquartinianum* A.Rich.	HE653603.1	*Campylosporus*
2	2	*Hypericumbalearicum* L.	AY555862.1	*Psorophytum*
3	3	Hypericumbellumsubsp.latisepalum N.Robson	HE653426.1	*Ascyreia*
	4	*Hypericumcalycinum* L.	HE653431.1	
	5	*Hypericumforrestii* (Chittenden) N. Robson	HE653476.1	
	6	*Hypericumhookerianum* Wight et Arn.	KC709450.1	
	7	*Hypericumkouytchense* Lévl.	FJ694210.1	
	8	*Hypericumlagarocladum* N. Robson	HE662703.1	
	9	*Hypericumpatulum* Thunb. ex Murray	FJ694214.1	
	10	*Hypericumpseudohenryi* N. Robson	KC709447.1	
	11	*Hypericumwilsonii* N. Robson	HE653658.1	
	12	*Hypericummonogynum* L.	HE653544.1	
4	13	*Hypericumgeminiflorum* Hemsl.	HM162838.1	*Takasagoya*
5	14	*Hypericumandrosaemum* L.	KC709337.1	*Androsaemum*
	15	*Hypericumgrandifolium* Choisy	KC709385.1	
	16	Hypericum×inodorum Mill.	HE653565.1	
6	17	*Hypericumxylosteifolium* N. Robson	HE653659.1	*Lnodora*
7	18	*Hypericumprzewalskii* Maxim.	JF976672.1	*Roscyna*
8	19	*Hypericumbupleuroides* Griseb.	HE653429.1	*Bupleuroides*
9	20	*Hypericumattenuatum* Choisy	HE662752.1	*Hypericum*
	21	*Hypericumkamtschaticum* Ledeb.	HE653516.1	
	22	*Hypericumperforatum* L.	JN811136.1	
	23	Hypericumperforatumsubsp.veronense (Schrank) H. Lindb.	MN036448.1	
	24	*Hypericumpseudopetiolatum* R. Keller	AY573002.1	
	25	*Hypericumyezoense* Maxim.	AY573004.1	
10	26	*Hypericumconcinnum* Benth.	HE653442.1	*Concinna*
11	27	*Hypericumpseudomaculatum* Bush	HE653595.1	*Graveolentia*
12	28	*Hypericumsampsonii* Hance	HE653620.1	*Sampsonia*
13	29	*Hypericumelodeoides* Choisy	HE653457.1	*Elodeoida*
14	30	*Hypericummonanthemum* Hook. f. et Thoms. ex Dyer	HE653542.1	*Monanthema*
15	31	*Hypericumpolyphyllum* Boiss. & Balansa	HE662730.1	*Olympia*
16	32	*Hypericumcerastoides* (Spach) N.Robson	AY555884.1	*Campylopus*
17	33	*Hypericumpapillare* Boiss. & Heldr.	HE653570.1	*Origanifolia*
18	34	*Hypericumbarbatum* Jacq.	FJ694192.1	*Drosocarpium*
	35	Hypericumricherisubsp.grisebachii (Boiss.) Nyman	FJ694222.1	
	36	*Hypericumrumeliacum* Boiss.	HE653616.1	
19	37	*Hypericumhumifusum* L.	HE653507.1	*Oligostema*
20	38	*Hypericumorientale* L.	HE653565.1	*Crossophyllum*
21	39	*Hypericumpseudolaeve* N.Robson	HE653594.1	*Hirtella*
22	40	*Hypericumhirsutum* L.	HE653500.1	*Taeniocarpium*
	41	*Hypericumpulchrum* L.	FJ694219.1	
23	42	*Hypericumempetrifolium* Willd.	HE653464.1	*Coridium*
24	43	*Hypericumhypericoides* (L.) Crantz	KC709376.1	*Myriandra*
	44	*Hypericumkalmianum* L.	FJ694209.1	
	45	*Hypericumprolificum* L.	MT551029.1	
25	46	*Hypericumcanariense* L.	KC709387.1	*Webbia*
26	47	*Hypericumvacciniifolium* Hayek & Siehe	HE653656.1	*Arthrophyllum*
27	48	*Hypericumpallens* Banks & Sol.	AY555848.1	*Triadenioides*
28	49	*Hypericumheterophyllum* Vent.	HE653492.1	*Heterophylla*
29	50	Hypericumaegypticumsubsp.webbii L.	KC709380.1	*Adenotrias*
30	51	*Hypericumpapuanum* Ridl.	HE653571.1	*Humifusoideum*
31	52	*Hypericumreflexum* L.f.	HE662747.1	*Adenosepalum*
32	53	*Hypericumelodes* L.	FJ694200.1	*Elodes*
33	54	*Hypericummexicanum* L.	LT904662.1	*Brathys*
34	55	*Hypericumjaponicum* Thunb. ex Murray	HE653513.1	*Trigynobrathys*
Outgroup		*Thorneacalcicole* Standl. & Steyerm	AY573028.1	–

The sequences were imported into BioEdit 7.0 for manual alignment and sorting, resulting in a refined sequence matrix, which was then exported (Hall 1999). The ITS matrix (Miller et al. 2010) was imported into the CIPRES supercomputer, and the optimal tree model was constructed using the appropriate Bayesian method (Ronquist and Huelsenbeck 2003; Ruchisansakun et al. 2016). The most suitable alternative model for base evolution was chosen based on the Bayesian Information Criterion (BIC). For the ITS sequence of nuclear genes, the GTR+I+G model was identified as the best model. The successful construction of the phylogenetic tree was achieved by analysing the ITS dataset using Bayesian inference (BI).

### ﻿Morphology

During the period of 2022–2023, we conducted a field investigation on *H.liboense* in Maolan National Nature Reserve, Guizhou Province, including photographing its characteristics and collecting seven live specimens. The type specimen is deposited in the Tree Herbaria, College of Forestry, Guizhou University, Huaxi District, Guiyang City, Guizhou Province, China (GZAC, GZAC–LB–0001). The morphological comparison of *H.liboense* specimens with similar species, such as *H.monogynum*, was conducted by studying various materials including leaves, flowers, fructus, and branches. This comparison was primarily based on authoritative plant literature, specifically descriptions found in Flora of China (Li et al. 1990) and Flora of Guizhou (Li 1986). Additionally, the identification process involved referencing sample images from the website (http://plants.jstor.org/) and detailed plant morphological descriptions available at Plant Information System (http://www.iplant.cn/). After measuring the traits with Vernier callipers, the data were analysed and compared with those from specimens of similar species. We directly collected fresh leaf materials in the field, placing them into FAA fixation solution.

## ﻿Results

**Figure 1.**
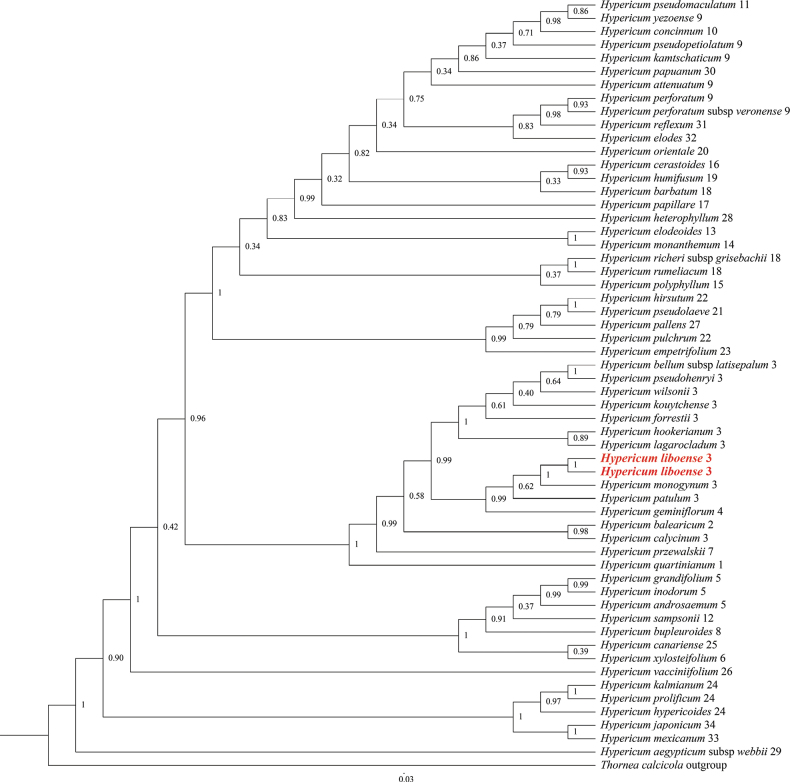
Partial Bayesian consensus phylogram based on ITS sequences. Numbers above branches are Bayesian posterior probabilities (The number after the species name represents the section of *Hypericum*; Table 1).

**Figure 2.**
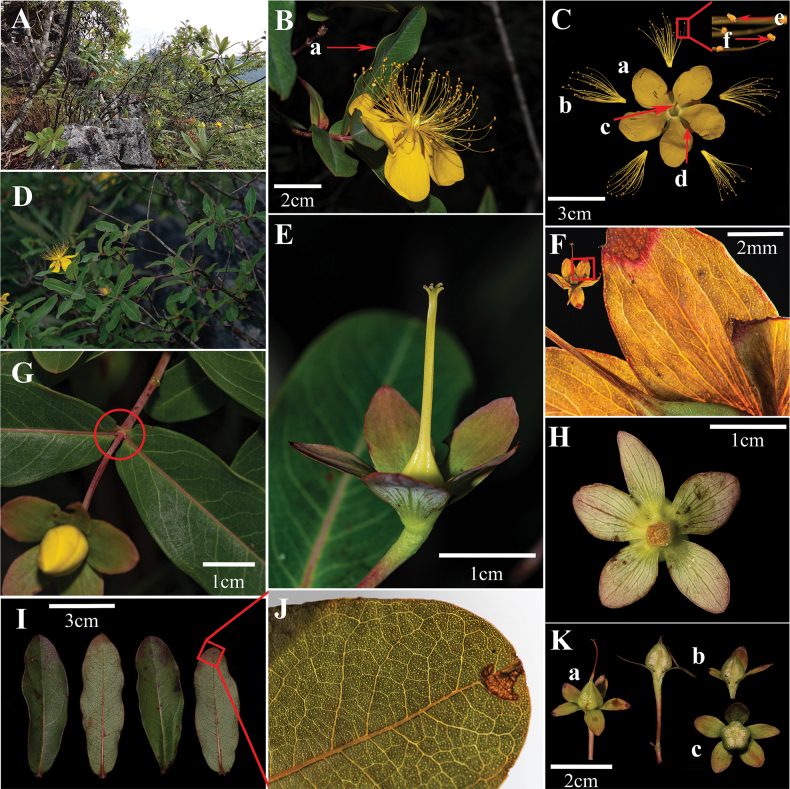
*Hypericumliboense***A** habitat **B** flower (a) undulating leaf margins **C** flower anatomy (a) petal (b) stamens (c) pistil (d) calyx (e) anther (f) gland **D***H.liboense* branch with flowers **E** stylus **F** veins and glandular points of calyx **G** leaf blade half-clasping twig **H** abaxial side of calyx **I** blade **J** veins and glands **K** anatomy of fructus (a) whole fructus (b) longitudinal section of fructus (c) cross-section of fructus

Phylogenetic analyses indicated that the 34 included taxa of *Hypericum* formed a well-supported monophyletic group (Fig. 1). Two individuals of the inferred new species from the sites in Libo County were resolved as a strongly-supported monophyletic lineage (PP = 1.00), which further clustered with *H.monogynum*, *H.patulum* and *H.geminiflorum* into a subclade (PP = 0.99). The tree shows that *H.liboense* is the sister species of *H.monogynum*, with relatively strong support (PP = 1.00). These two species also showed certain morphological similarities, especially those of the petals, stamens and pistils (Fig. 2; Table 2). *H.patulum* has the closest relationship to *H.liboense* and *H.monogynum*, although with relatively poor support (PP = 0.62), followed by *H.geminiflorum* (PP = 0.99).

**Table 2.** Morphological comparison of *Hypericumliboense* and similar species.

	*H.liboense*	*H.monogynum*	*H.kouytchense*	*H.patulum*
Petiole	Leaves sessile, semi-amplexicaul	Leaves sessile or brachy petiolate	Leaves petiolate, 0.5–1.5 mm long	Leaves petiolate, 0.5–2 mm long
Leaf texture	Thickly papery to thinly leathery	Thickly papery	Thickly papery	Thickly papery
Leaf morphology	Long elliptical to long circular	Oblanceolate or elliptic to long circular	Elliptic, lanceolate to ovate or triangular ovate	Blade lanceolate or oblong-lanceolate to ovate or oblong-ovate
Leaf margin	Slight undulation	Flat	Flat	Flat
Leaf lower epidermis character	Greyish white with white powder	Light green, not grey	Light green, not grey	Abaxially rather glaucous
Midvein	Midvein raised on both sides of the leaf surface	Midvein flat on the leaf surface	Midvein flat on the leaf surface	Midvein flat on the leaf surface
Main lateral veins	8–15-paired	4–6-paired	3–4-paired	3-paired
Tertiary reticulation	Conspicuous, sunken on the leaf surface	Not very conspicuous	Obscure or invisible	Scarcely visible
Inflorescence	1–3-flowered	1–15(–30)-flowered	1–7(–11)-flowered	1–15-flowered
Anther	Yellow, glandular	Yellow to dark orange, glandular	Yellow, glandular	Yellow, glandless
Calyx size	Elliptic or broad ovate, larger, 10–14×4–6 mm	Broad or narrowly elliptic or oblong to lanceolate or oblanceolate, smaller, 4.5–13×1.2–2 mm	Oblong-ovate to lanceolate, larger, 7–15×2.5–7 mm	Broadly ovate or broadly elliptic or subcircular to oblong-elliptic or obovate-spatulate, 5–10×3.5–7 mm
Calyx margin	Margin entire	Margin entire	Margin entire	Margin eroded-denticulate to ciliolate with markedly hyaline margin
Thin veins of calyx	Obvious	Obscure	Obscure	Obscure

## ﻿Discussion

The results of our phylogenetic tree showed that all species of sect. Asian (including *H.patulum*, *H.kouytchense* and *H.monogynum*, etc) clustered together into a single clade with strong support (PP = 1.00), and the results were consistent with previous studies (Nürk et al.2013; Meseguer et al. 2014). In the phylogenetic tree the new species and *H.monogynum* were placed along with the species of sect. Asian. The phylogenetic trees (ITS; Fig. 2) indicate that *H.liboense* is a distinct member of *Hypericum*, and furthermore, support its sister taxon relationship with *H.monogynum*, thus corroborating the evidence provided by the morphological observations. There are two populations of *H.liboense* that have been recorded and observed, and we find that the morphological characters of the species present consistency between the two populations, especially with respect to the morphology of leaves, inflorescence, and calyx. *H.liboense* is similar to *H.monogynum* in having whole leaf margins, glandular, yellow petals, whole and flat calyx margins, stamen fascicles each with 25–35 stamens, styles united nearly to apices, then out curved free. but differs from *H.monogynum* in that the leaf edges are wavy (vs. flat), the midvein are raised on both sides (vs. raised on lower epidermis of leaves), the tertiary venation is sunken on both surfaces (vs. surface not sunken), and the abaxial leaf surface are greyish-white (vs. without grey), inflorescences with 1–3 flowers (vs. 1–15(–30) flowers), calyx are elliptic or broad-ovate (vs. broad or narrowly elliptic or oblong to lanceolate or oblanceolate), and veins obvious (vs. obscure), wholly punctiform glands (vs. laminar glands basally lines to streaks). This feature of wavy leaf edges, obvious veins of leaves and calyx, inflorescence 1–3-flowered is crucial for distinguishing *H.liboense* from *H.monogynum* and other related species (Table 2), and supports its standing as a separate, and new species. Furthermore, to our knowledge, most calyx of *Hypericum* species have obscure thin veins; there is also no report of variation in the leaf edges of *H.liboense*. Therefore, we believe that the wavy leaf edges and distinctly thin veined of calyx are reliable trait for this purpose and that the recognition of *H.liboense* as a new species is strongly supported (PP = 1.00) by ITS phylogenetic tree.

In recent years, new species of the genus Hypericaceae have been gradually discovered and reported (Ocak et al. 2009; Bacchetta et al. 2010; Tan et al. 2010; Ely and Boldrini 2015; Ely et al. 2015; Marinho et al. 2016; Trigas 2018; Galindon et al. 2021), indicating the important potential role of discovering and documenting new species in enriching regional species diversity and prioritizing conservation efforts in biodiversity hotspots. As time progresses, the discovery of new species is increasingly receiving attention. The discovery location of *H.liboense* is in the karst landscape-rich area of Guizhou Province, China. The discovery and description of this species further highlight the ability of the karst limestone region to support rich species diversity and endemism, while also providing favourable conditions for the survival of *H.liboense*. Therefore, conducting a comprehensive investigation and study of the phylogeny and morphology of *H.liboense* in the karst limestone region of southern China will provide important scientific insights into the plant diversity and the implementation of conservation strategies in this region.

### ﻿Taxonomy

#### ﻿ Hypericum liboense

Taxon classificationPlantaeMalpighialesHypericaceae

M.T.An & T.R.Wu sp. nov.

B3BBA475-295B-5C93-A944-E8145B5069AA

urn:lsid:ipni.org:names:77334341-1

Fig. 2

##### Type.

China, Guizhou Province, Libo County, Maolan National Nature Reserve, elev. 947, 25°16'N, 107°57'E, 21 April 2022, Jian Xu, Mingtai An and Tianrou Wu 220421(Holotype: GZAC–LB–0001, Isotype: GZAC–LB–0002).

##### Diagnosis.

This species is similar to *H.monogynum* in terms of morphology. The main difference between the two species is that the leaves of *H.liboense* are sessile and semi-clasped (vs. leaves sessile or brachypetiolate). The leaves of *H.liboense* are long elliptical to long circular, and the edges are whole and wavy (vs. oblanceolate or elliptic to long circular, flat). *H.liboense* leaves are thickly papery to thinly leathery (vs. thickly papery), with a white powder on the abaxial side leading to a grey-white appearance (vs. abaxially without grey). Main lateral veins of leaves 8–15 pairs (vs. 4–6 pairs), with the midvein on both sides convex, the main lateral veins obvious branches from the midvein, the main lateral veins and tertiary vein forming an obvious network and obviously sagging (vs. tertiary vein obscure and not sunken). Inflorescences with 1–3 flowers (vs. 1–15(–30) flowers), calyx are elliptic or broad-ovate (vs. broad or narrowly elliptic or oblong to lanceolate or oblanceolate), 10–14 mm long, 4–6 mm wide (vs. 4.5–13 mm long and 1.2–2 mm wide), and veins obvious (vs. obscure) (Table 2).

##### Description.

***Plants*** Erect shrub, 0.5–1.3 m tall. Young branches reddish brown with a light white powder. Old branches dark reddish-brown or grey, cylindrical, with a lumpy rind after cracking off, and the cortex light red. ***Leaves*** opposite, sessile, with semi-clasping branchlets. The leaves are long elliptical to long circular, 4–8 cm long and 2–4 cm wide, with the middle entire part of the leaf usually the widest, the apex blunt round, with a fine cusp; leaf blade base cuneate to rounded, margin entire and slightly ruffled; thickly papery to thinly leathery, glabrous, the surface of the leaves green or dark green, the back of the leaves white and greyish-white; the main lateral veins of the leaves in 8–15 pairs, the midvein raised on both sides, and the base reddish; main lateral veins and midvein branching obviously, main lateral veins and tertiary vein forming an obvious network and concave on the leaf surface; wholly punctiform glands. ***Inflorescence*** with 1–3 flowers, emanating from the first segment of the stem; ***peduncle*** yellow-green, 1.3–3 cm long. ***Flowers*** 4–7 cm in diameter; bud ovular, apex subacute. ***Calyx*** 5, free, ovate to broadly ovate, 1–1.4 cm long, 0.4–0.6 cm wide, wholly punctiform glands, apex acute to rounded, entire margin, base light green, margin purplish red, midvein and veining obvious, and calyx enlarged in the fruit stage. ***Petals*** 5, yellow, without flush, open, triangular obovate, slightly curved, 2.8–3.5 cm long, 1.6–2.2 cm wide, approximately, margin entire, glandular. ***Stamens*** in 5 fascicles, each with 23–40 stamens, 1.3–3.4 cm long, several times the length of the petal, anthers yellow to dark orange, with glands. ***Ovary*** ovulate or sub-globular, 3–5 mm long, 2.5–5 mm wide. ***Style*** 1.3–2.2 cm long, styles partly united (style confluent almost to apex and then curved outwards into 5 splits), stigma small, lavender. ***Capsule*** broadly oval-shaped or oval-shaped and conical, 10–14 mm long and 6–10 mm wide, light green, dark brown when ripe.

##### Phenology.

Flowering from April to June; fruiting from June to September.

##### Distribution and ecology.

This species is known to be found only in Libo County, Guizhou Province, China, on the top of a mountain in a karst landscape, alt. 947 m.

##### Etymology.

The species name “liboense” refers to the origin of the type specimen, Libo County, Guizhou Province.

##### Conservation status.

In the 2022–2023 period, we sampled the *H.liboense* population and found two more sites around the location where the species was first discovered, each with a population of approximately 20 plants. The habitat of *H.liboense* is mainly from the exposed rock gully area above the middle of the mountain to the top of the mountain. The soil in the plant habitat is poor, the soil layer is weak in its ability to retain water, and drought is common. At present, *H.liboense* is not known to be distributed in the low-altitude areas below the foot of the mountain and the middle of the mountain, so we hypothesize that the current availability of habitat for *H.liboense* is relatively poor and the population is relatively endangered. However, because our current investigation of the survival status and threat factors of *H.liboense* is not sufficiently comprehensive to provide information on the specific distribution of this population, we recommend that *H.liboense* be classified as “data missing” (IUCN 2017).

## ﻿Conclusions

According to the morphological characteristics and molecular evidence of *Hypericum*, the findings indicate that *H.liboense* should be categorized within sect. Ascyreia. It is evident that *H.liboense* is a distinct member of *Hypericum* and forms a strongly supported clade (PP = 1.00) within the *Hypericum* phylogenetic tree. Moreover, *H.liboense* exhibits distinct morphological features that differentiate it from all currently accepted species in *Hypericum*. Therefore, it is deemed necessary to classify *H.liboense* as a new species.

## Supplementary Material

XML Treatment for Hypericum liboense

## ﻿Additional information

### Author ORCIDs

Tian-Rou Wu https://orcid.org/0009-0001-5223-0567

Jian Xu https://orcid.org/0000-0002-0714-0917

Ming-Tai An https://orcid.org/0000-0003-3886-0287

Jiang-Hong Yu https://orcid.org/0000-0003-1765-8557

Feng Liu https://orcid.org/0009-0003-7031-4494

### Data availability

All of the data that support the findings of this study are available in the main text.
